# Silymarin as a phytopharmaceutical agent: advances in mechanistic insights, formulation strategies, and pre-clinical applications

**DOI:** 10.3389/fphar.2025.1711653

**Published:** 2025-11-27

**Authors:** Mahewish Sayyad, Ashish Dilip Sutar, Kamini Shivhare, Rahul Shukla, Swaran J. S. Flora

**Affiliations:** 1 Department of Biotechnology, Priyadarshini College of Engineering, Nagpur, India; 2 Department of Pharmaceutics, National Institute of Pharmaceutical Education and Research (NIPER, Raebareli), Raebareli, India; 3 Department of Pharmacy, Era Medical College and University, Lucknow, India

**Keywords:** silymarin, herbal moiety, neuro-regenerative medicine, immunomodulator, nanocarrier, cellular pathway

## Abstract

Silymarin, extracted from the seeds of Silybum marianum (milk thistle), has been utilised in traditional medicine for many years and is recognised for its neuroprotective and hepatoprotective properties. Existing research reveals that silymarin has potent antioxidant and anti-inflammatory effects, coupled with anti-fibrotic, anti-carcinogenic, neuro-regenerative, and immunomodulatory actions, and has broad therapeutic relevance in both neurological and hepatic disorders, albeit with the drawback of low solubility. Silymarin participates in multiple molecular and cellular pathways to show its therapeutic effect. Nanocarriers are a promising solution to silymarin’s low solubility and limited bioavailability, aiming to enhance targeted delivery to the central nervous system and hepatic tissue. These developments position silymarin as a multi-domain phytopharmaceutical with significant translational potential. This review provides an overview of silymarin’s historical context, phytochemical composition, and pharmacokinetic profile, with a particular focus on targeted drug delivery systems. Special emphasis is placed on its potential role in managing various diseases.

## Graphical Abstract

**Figure:**
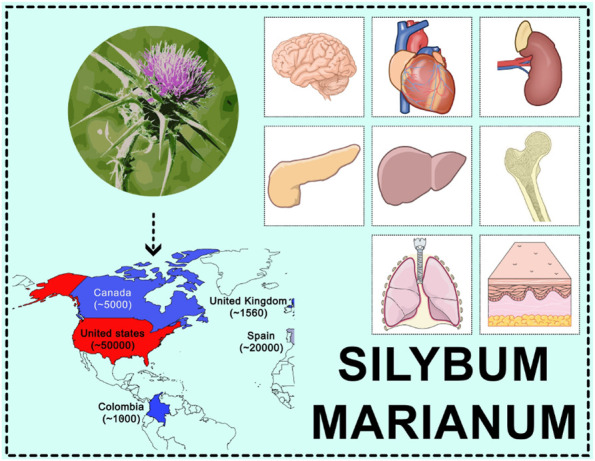

## 1 Introduction

Natural compounds have gained significant momentum as a therapeutic approach in contemporary pharmacology, offering a multi-targeted intervention with low safety risks. Among them, silymarin, a phytocomplex of *Silybum marianum* (milk thistle), has emerged as one of the most mechanistically characterised botanical agents in hepatoprotective and neuroprotective therapy. Silymarin has a rich history of use in traditional medicine, and its recognised hepatoprotective, neuroprotective and antioxidant effects are now understood in relation to its complex phytochemical composition and the downstream molecular pathways it modulates (Wadhwa et al., 2022; Akhtar et al., 2023; Shivaprasad et al., 2025). Mechanistic studies have clarified that silymarin’s major bioactive constituents, like silibinin, silydianin, and silychristin, interact with redox-sensitive transcriptional pathways such as Nrf2/ARE and NF-κB, while also influencing metabolic regulators like AMPK and SIRT1 (Li et al., 2019a; Lou et al., 2024). These mechanisms collectively work for their antioxidant, anti-inflammatory, and antifibrotic actions. Moreover, advances in pharmacokinetic profiling have highlighted the extensive first-pass metabolism and low oral bioavailability of this compound, prompting the development of novel formulation strategies, including nanoemulsions, liposomes, phospholipid complexes, and polymeric nanoparticles (Hu et al., 2023; Mathure et al., 2023; Rizwan et al., 2023; Lou et al., 2024; Behera et al., 2025). This review offers a modern interpretation integrating silymarin’s historical, biochemical, and pharmacological view with current insights into bioavailability enhancement and mechanistic targeting, providing a translational perspective for its clinical and formulation development (Sharma et al., 2024; Tolangi et al., 2024; Zhang et al., 2024).

### 1.1 Historical significance of silymarin

Therapeutic properties of milk thistle were first documented by the Greek physician and botanist Dioscorides (in 40–90 AD). Later, in 1597, John Gerard recognised it as one of the most effective remedies for depressive disorders. Theophrastus (371–287 BC) was the first to mention milk thistle as Pternix, showing its long history of medicinal use. Both Pliny the Elder and Dioscorides described its uses in their works. By the 16th century, milk thistle was an effective remedy for liver and gallbladder disorders. In his book, Nicholas Culpeper noted silymarin’s effectiveness for treating blockages in the spleen and liver. Early European colonists brought milk thistle to the Americas. By the early 20th century, herbalists used it to treat issues related to the kidneys, liver, spleen, and menstruation. Interest in its healing properties continued until the 1960s, when German research renewed focus on its ability to treat liver disorders and protect the liver from harmful toxins. By the late 19th and early 20th centuries, herbalists were specialising in its treatment for various ailments, including liver, spleen, kidney, and reproductive system disorders, with a focus on irregular menstruation. This extensive ethnomedical history laid the foundation for silymarin’s modern mechanistic evaluation as a polyphenolic antioxidant complex influencing hepatic detoxification and mitochondrial redox regulation (Li et al., 2019a; Hu et al., 2023).

### 1.2 Botanical source

**FIGURE 1:**
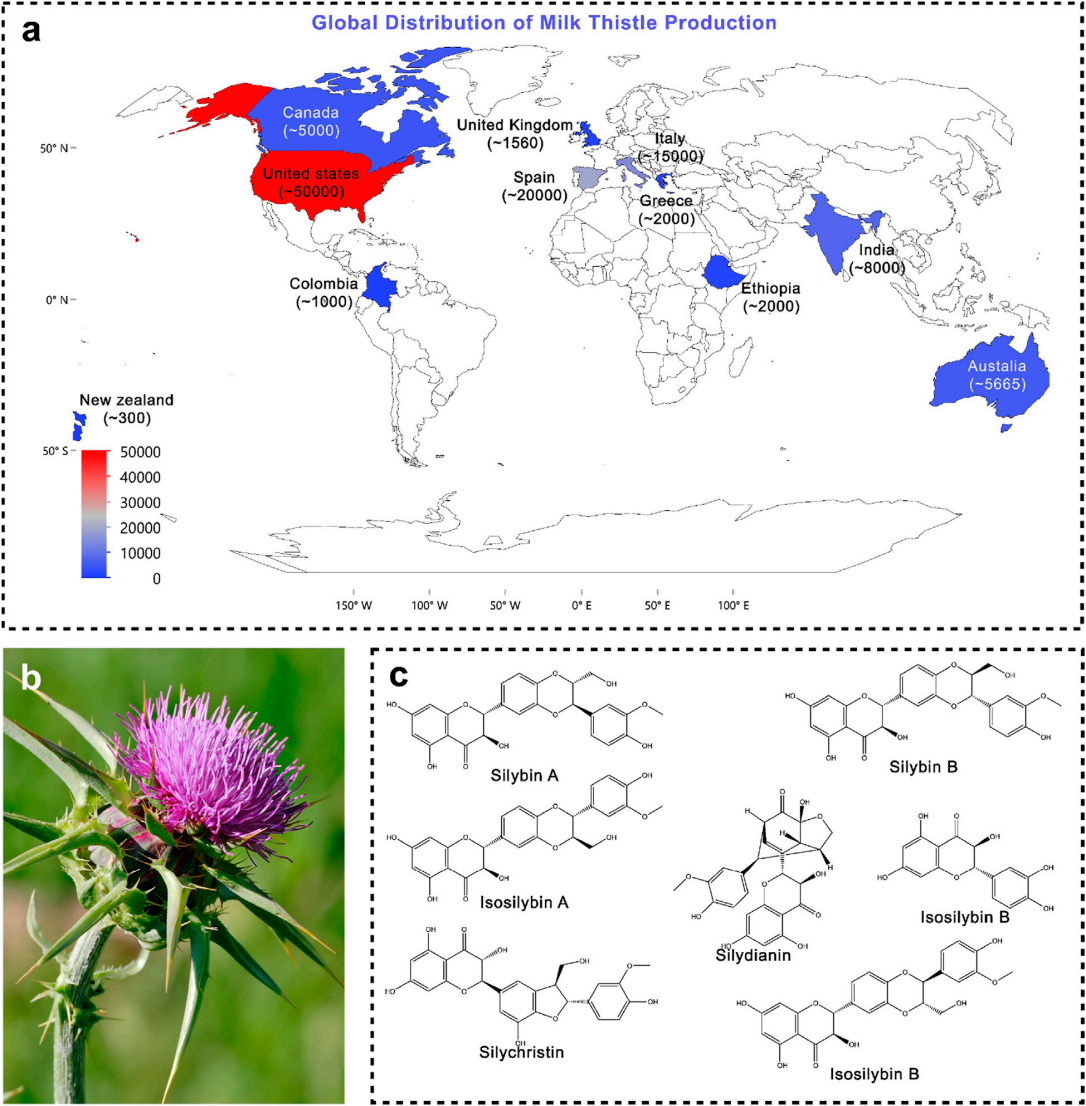
**(a)** Global production of silymarin; **(b)** Morphological representation of *Silybum marianum* (photograph) (Source: https://en.wikipedia.org/wiki/Silybum_marianum); **(c)** Major phytoconstituents of *Silybum marianum*.

*Silybum marianum* (L.) Gaertn. (syn. *Carduus marianus* L.), commonly known as milk thistle, is also referred to by various other names, including blessed milk thistle, blessed virgin thistle, Christ’s crown, heal thistle, holy thistle, Marian thistle, Mary thistle, Saint Mary’s thistle, our lady’s thistle, sow thistle, variegated thistle, Venus’s thistle, and wild artichoke. Milk thistle is a wild, thorny plant belonging to the family Asteraceae. It typically grows as an annual species, though in rare cases it may persist as a biennial. Owing to its rapid, competitive, and invasive growth, it is often regarded as a weed in many regions. The plant generally attains a height of 90–200 cm, but under favourable conditions it can grow as tall as 300 cm (Karkanis et al., 2011; Andrzejewska et al., 2015). Milk thistle is characterised by its distinctive morphology, which makes it easily identifiable among members of the Asteraceae family. The plant bears large, solitary, globular purple flower heads surrounded by sharp, spiny bracts, which serve as a natural defence against herbivores (Figure 1b). Its broad, oblong to lanceolate leaves are glossy green with striking milky-white marbling along the veins, giving rise to the common name “milk thistle.” The leaf margins are strongly serrated and armed with stiff spines, further adding to its thorny appearance. The fruit is a smooth, shiny black achene, equipped with a whitish, oily appendage known as an elaiosome. This structure plays a crucial role in myrmecochory seed dispersal mediated by ants, thereby enhancing the plant’s ecological adaptability (Gresta et al., 2006). Geographically, *S. marianum* is native to the Mediterranean basin, where it has a long history of both ecological presence and medicinal use. However, due to its robust adaptability and efficient seed dispersal mechanisms, it has naturalised far beyond its native range. Today, the species is widely distributed across central and southern Europe, North and South America, South Africa, and Australia, often thriving in disturbed soils, roadsides, pastures, and agricultural fields. In many regions, it is considered an invasive weed due to its vigorous growth, competitive ability, and capacity to form dense stands that outcompete native vegetation (Gresta et al., 2006). Despite this, its medicinal significance has ensured its continued cultivation and use on a global scale (Figure 1a shows production of silymarin in tons per year). The taxonomic identity of *S. marianum* (L.) *Gaertn.* has been verified using the Medicinal Plant Names Services (MPNS) database, confirming its validity under the family Asteraceae (Heinrich et al., 2022; Royal Botanic Gardens, 2025). Recent agronomic and genetic studies suggest that phytochemical yield and flavonolignan ratios are significantly influenced by geographic and climatic variables, which can impact both therapeutic potency and formulation stability (Hu et al., 2023; Sun W. et al., 2025).

### 1.3 Traditional and modern applications of milk thistle

For centuries, silymarin has been used extensively in traditional medicine throughout Europe and beyond. Given its long history of being linked to liver health, it has historically been prescribed for jaundice and a number of hepatobiliary conditions (Federico et al., 2017). In cases of food poisoning caused by hepatotoxic fungi, particularly Amanita species, preparations of milk thistle seeds were utilised as an antidotal remedy. Various plant parts were used for a variety of therapeutic purposes in traditional European folk medicine. The aerial parts were advised for uterine disorders, dropsy, and intermittent fevers (Emadi et al., 2022). The plant’s leaves were prized for their diaphoretic and digestive-promoting qualities, while extracts were also used externally to treat cancers. Rich in active phytoconstituents, the seeds were used to treat bleeding, lessen irritation, and ease spasms. They were also known to have hepatoprotective properties. It has been reported that alcoholic seed extracts act as a mild purgative by increasing intestinal peristalsis (Emadi et al., 2022; Marceddu et al., 2022).

In the modern era, Silymarin exhibits potent antioxidant and anti-inflammatory properties. It stabilises cellular membranes, enhances glutathione levels, scavenges reactive oxygen species (ROS), and modulates inflammatory pathways (e.g., NF-κB, Nrf2) (de Freitas et al., 2024; Dhande et al., 2024; Tahmasbi et al., 2024). Recent mechanistic evidence suggests that these pathways converge on mitochondrial protection and the regulation of oxidative phosphorylation, contributing to their dual hepatocellular and neuroprotective actions (Lou et al., 2024). Silymarin has shown protective effects in chronic liver diseases, such as cirrhosis, hepatitis, and non-alcoholic fatty liver disease (NAFLD), improving liver function and histological outcomes (de Freitas et al., 2024). Although there is conflicting human data, preclinical models support its function in reducing liver damage from toxins and chemotherapy. According to clinical research, silymarin may help people with type 2 diabetes lower their blood sugar levels and insulin resistance, particularly when combined with conventional treatment (Dhande et al., 2024). *In vitro* and in animal models, it has demonstrated chemo preventive effects by preventing the growth of different cancer cell lines and encouraging apoptosis. It may also mitigate the toxicity caused by chemotherapy (Emadi et al., 2022). According to preliminary research, silymarin exhibits anti-inflammatory and antioxidant properties that may aid in treating inflammatory and oxidative eye disorders. Aldose reductase and VEGF inhibition are examples of specific mechanisms, but further study is needed (Tahmasbi et al., 2024). Beyond its pharmacodynamic profile, novel drug delivery systems have been shown to significantly enhance oral absorption and systemic exposure, thereby enabling a more effective translation of their mechanistic benefits to clinical efficacy. Furthermore, genetic and metabolic engineering efforts are directed toward enhancing flavonolignan biosynthesis, optimising industrial-scale production, and ensuring consistency in therapeutic formulations.

## 2 Phytochemistry of *Silybum marianum*

Silymarin is a complex mixture of four flavonolignan isomers, i.e., silybin, isosilybin, silydianin, and Silychristin (Wagner et al., 1974). In addition to flavonolignans, the fruit of milk thistle contains a number of other bioactive substances, such as flavonoids (taxifolin [C_15_H_12_O_7_], quercetin [C_15_H_10_O_7_], dihydrokaempferol, kaempferol [C_15_H_10_O_6_], apigenin [C_15_H_10_O_5_], naringin, eriodyctiol, and chrysoeriol), 5,7-dihydroxychromone, dehydroconiferyl alcohol, fatty acids (58%–60% linoleic acid, 30% oleic acid, 8.5%–9% palmitic acid), vitamin E derivatives (tocopherols), sterols (cholesterol, campesterol, stigmasterol, and sitosterol), sugars (arabinose and glucose), and small peptides (Abenavoli et al., 2010). Among these, silybin constitutes the major active component, representing about 50%–70% of the total silymarin extract, and is considered the principal biomarker (Luper, 1998; Abenavoli et al., 2010). A degradative method was used in 1975 to determine the absolute stereochemistry at the C-2 and C-3 positions. It is also known by alternative names such as flavobin, silybine, silliver, and silybina. Its molecular formula is C_25_H_22_O_10_, with a molecular weight of 482.44 g/mol, and it is registered under CAS No. 22888-70-6 (Pelter and Hänsel, 1968).

In terms of structure, silybin is made up of two subunits connected by an oxeran ring: a flavanonol moiety derived from taxifolin and a phenylpropanoid unit derived from coniferyl alcohol. Three of the molecule’s five hydroxyl groups—5-OH, 7-OH, and 20-OH—are phenolic. These hydroxyl groups are essential to silybin’s derivatisation and reactivity because they engage in hydrogen bonding. Because of its lower steric hindrance, the C-7 hydroxyl group is more reactive than the C-20 hydroxyl group. While the C-3 hydroxyl group readily oxidises to a ketone, forming 2,3-dehydrosilybin, the C-23 hydroxyl group permits esterification. When it comes to solubility, silybin is insoluble in nonpolar solvents, such as diethyl ether and chloroform, and is poorly soluble in polar protic solvents, like ethanol and methanol. However, polar aprotic solvents like acetone are good for dissolving it (Biedermann et al., 2014).

### 2.1 Bioactive compounds of milk thistle

The primary bioactive constituents of Silybum marianum (milk thistle, MT) (Figure 1c), collectively referred to as silymarin, are predominantly flavonolignans namely silybin A (PubChem CID: 31553), silybin B (PubChem CID: 1548994), isosilybin A (PubChem CID: 11059920), isosilybin B (PubChem CID: 10885340), silydianin (PubChem CID: 11982272), and silychristin (PubChem CID: 441764) alongside flavonoids such as taxifolin (PubChem CID: 439533) and quercetin (PubChem CID: 5280343), as well as other polyphenolic compounds. Among these, silybin (also known as silibinin) is recognised as the principal bioactive component, constituting approximately 50%–60% of total silymarin (Pelter and Hänsel, 1968; Luper, 1998; Lee et al., 2007; Abenavoli et al., 2010). Although silymarin is distributed throughout the plant, its highest concentration is localised in the seeds.

## 3 Molecular mechanism of silymarin

Silymarin have its neuroprotective and hepatoprotective effects through multiple interconnected molecular pathways (Table 1). The key pathways contributing to these therapeutic actions are outlined below.

**TABLE 1:** Molecular mechanisms underlying the pharmacological actions of silymarin.

Sr. No.	Mechanism	Molecular event	Silymarin’s influence	Impact on cellular redox state	References
1	Nrf2 Regulation (Non-stressed)	Nrf2 is continuously produced, bound by KEAP1, ubiquitinated by CUL3/RBX1, and degraded by proteasome in cytoplasm.	No direct intervention maintains basal state. Under basal conditions, it does not alter NF-κB	Nrf2 levels are low, and antioxidant gene expression is basal.	Vargas-Mendoza et al. (2020)
2	Oxidative Stress Sensing	ROS modify cysteine residues on KEAP1 (e.g., C155, C273, C288).	Silymarin, as an antioxidant, can directly reduce ROS, but also acts on KEAP1. Indirectly suppresses NF-κB activation, due to lowered ROS.	KEAP1 undergoes conformational change, releasing Nrf2. Reduced oxidative and inflammatory stress.	Surai (2015)
3	Nrf2 Activation and Translocation	Released Nrf2 is phosphorylated (e.g., by PKCγ) and translocates to the nucleus.	Silymarin promotes Nrf2 phosphorylation and nuclear translocation. It may also inhibit TGF-β/SMAD signalling by avoiding oxidative-fibrotic feedback.	Nrf2 accumulates in the nucleus, ready to activate gene expression. Strengthened antioxidant and antifibrotic signalling.	Vargas-Mendoza et al. (2020), Bello (2023)
4	DNA Binding and Gene Transcription	Nuclear Nrf2 forms heterodimers with MAF proteins, binds to Antioxidant Response Elements (AREs) in gene promoters.	Silymarin facilitates Nrf2-MAF binding to AREs. Reduce NF-κB DNA binding and pro-inflammatory cytokine production.	Upregulation of cytoprotective genes. Enhanced anti-oxidant and anti-inflammatory response.	Fernandes Veloso Borges et al. (2018)
5	Antioxidant Enzyme Production	Transcription of genes for HO-1, NQO-1, GCLC, G6PD, and enzymes for GSH synthesis.	Silymarin directly enhances the expression of these enzymes. HO-1 suppresses NF-κB-driven inflammation.	Increased production of key antioxidant and detoxification enzymes and cofactors.	Dhande et al. (2024)
6	Cellular Redox Balance Restoration	Enhanced antioxidant capacity counteracts ROS, reduces oxidative damage.	Silymarin leads to a robust and sustained antioxidant response.	Comprehensive protection against oxidative stress, inflammation, and fibrotic damage.	Cai et al. (2024)

### 3.1 Antioxidant activity

**FIGURE 2:**
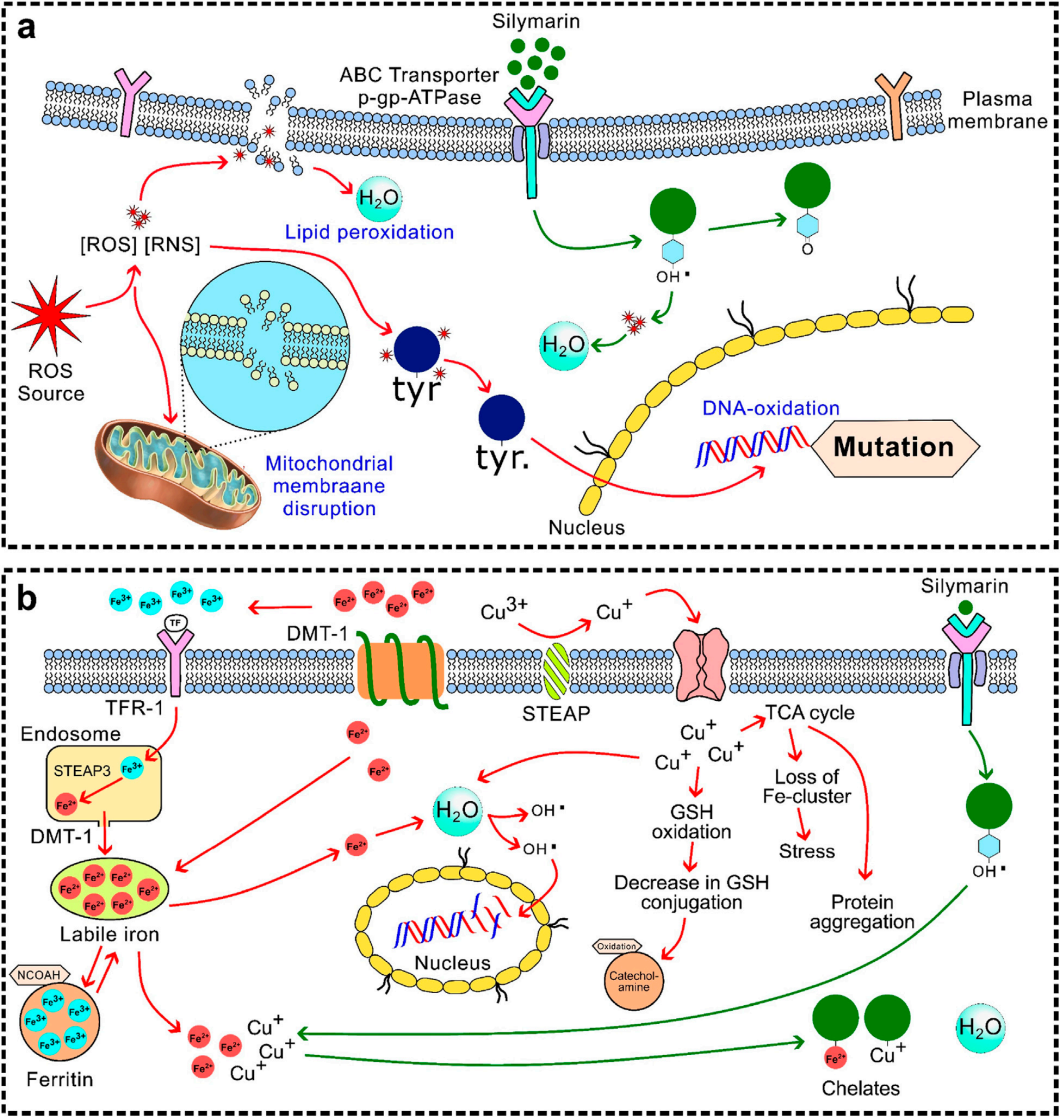
Antioxidant mechanisms **(a)** Free radical scavenging: Silymarin directly neutralise reactive oxygen species (ROS) and reactive nitrogen species (RNS), by preventing oxidative damage to lipids, proteins, and nucleic acids; **(b)** Metal chelation: Silymarin chelates the transition metal ions such as Fe^2+^ and Cu^2+^, inhibiting the generation of highly reactive hydroxyl radicals.

Silymarin, a flavonolignan complex, demonstrates strong antioxidant activity. In addition to exhibiting moderate activity against superoxide anions, its phenolic hydroxyl groups enable direct scavenging of reactive oxygen species (ROS), such as hydroxyl radicals and hypochlorous acid (Mahjoubin-Tehran et al., 2021; Jaffar et al., 2024). Silymarin works to provide broad-spectrum ROS neutralisation, which quickly lowers oxidative stress and stops cellular damage like protein oxidation, lipid peroxidation, and DNA strand breaks (Figure 2a) (Shaker et al., 2010). In addition to direct radical scavenging, silymarin preserves mitochondrial integrity by stabilising electron transport chain (ETC) complexes, reducing electron leakage, and inhibiting ROS-generating enzymes such as NADPH oxidase and xanthine oxidase (Surai, 2015; Taleb et al., 2018). This maintenance of mitochondrial function supports ATP production and overall cellular bioenergetics under oxidative stress (Ligeret et al., 2008).

**FIGURE 3:**
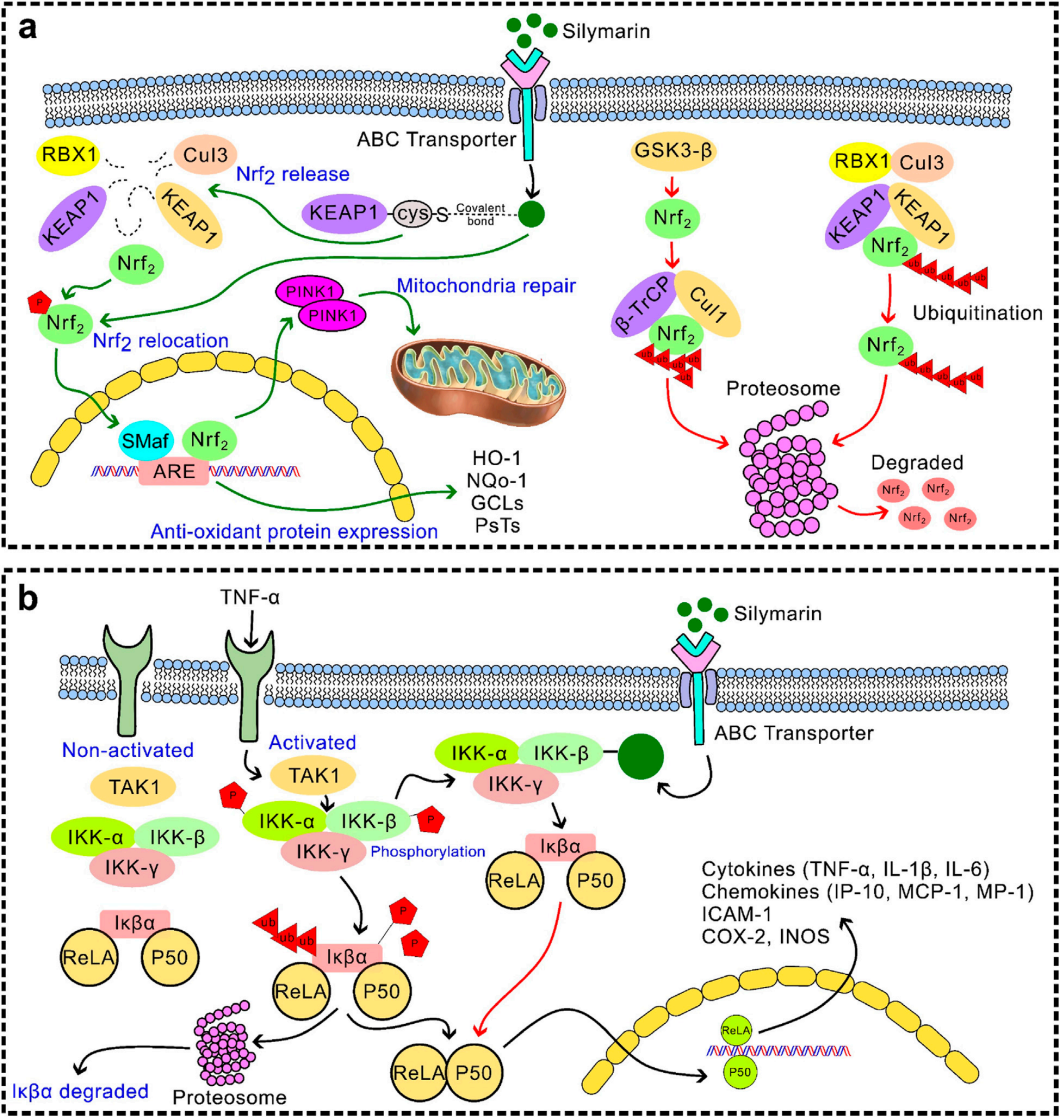
**(a)** Activation of the Nrf2 pathway: Silymarin enhances the nuclear translocation of nuclear factor erythroid 2-related factor 2 (Nrf2), leading to strengthening the cellular defence system against oxidative stress; **(b)** Inhibition of the NF-κB signalling pathway: Silymarin inhibits the phosphorylation and degradation of IκBα, by preventing nuclear translocation of NF-κB and reducing transcription of pro-inflammatory cytokines.

Silymarin enhances endogenous antioxidant defences by activating transcription factors Nrf2 (Figure 3a) and NF-κB (Figure 3b) (Reuland et al., 2013). Nrf2 translocation promotes the expression of antioxidant enzymes, including SOD, CAT, GPx, and HO-1, as well as phase II detoxifying enzymes. NF-κB modulation reduces the expression of pro-inflammatory cytokines and adhesion molecules, linking antioxidant and anti-inflammatory effects (Shaker et al., 2010; Kim et al., 2013; Surai, 2015). Additionally, silymarin increases vitagenes that improve cellular resilience, such as sirtuins, thioredoxin (Trx), and heat shock proteins (HSPs) (Trovato Salinaro et al., 2014; Dodson et al., 2019). As molecular chaperones, HSPs facilitate the breakdown of damaged proteins and guarantee correct protein folding (Demir et al., 2014; Hu et al., 2022). Trx regulates transcription factors implicated in stress responses and preserves the redox state of proteins. Sirtuins improve cellular survival under stress by controlling mitochondrial biogenesis, DNA repair, apoptosis, and metabolic efficiency (Demir et al., 2014; Jiao et al., 2021). Silymarin contributes to antioxidant defence through metal chelation by binding transition metals such as Fe^2+^ and Cu^2+^, thereby inhibiting ROS generation *via* the Fenton reaction (Figure 2b) (Abe et al., 2022). In hepatocyte culture studies, pretreatment with silymarin significantly attenuated Fe^2+^-induced intracellular ROS accumulation, reducing it by approximately 52% (Gillessen and Schmidt, 2020).

### 3.2 Anti-inflammatory activity

**FIGURE 4:**
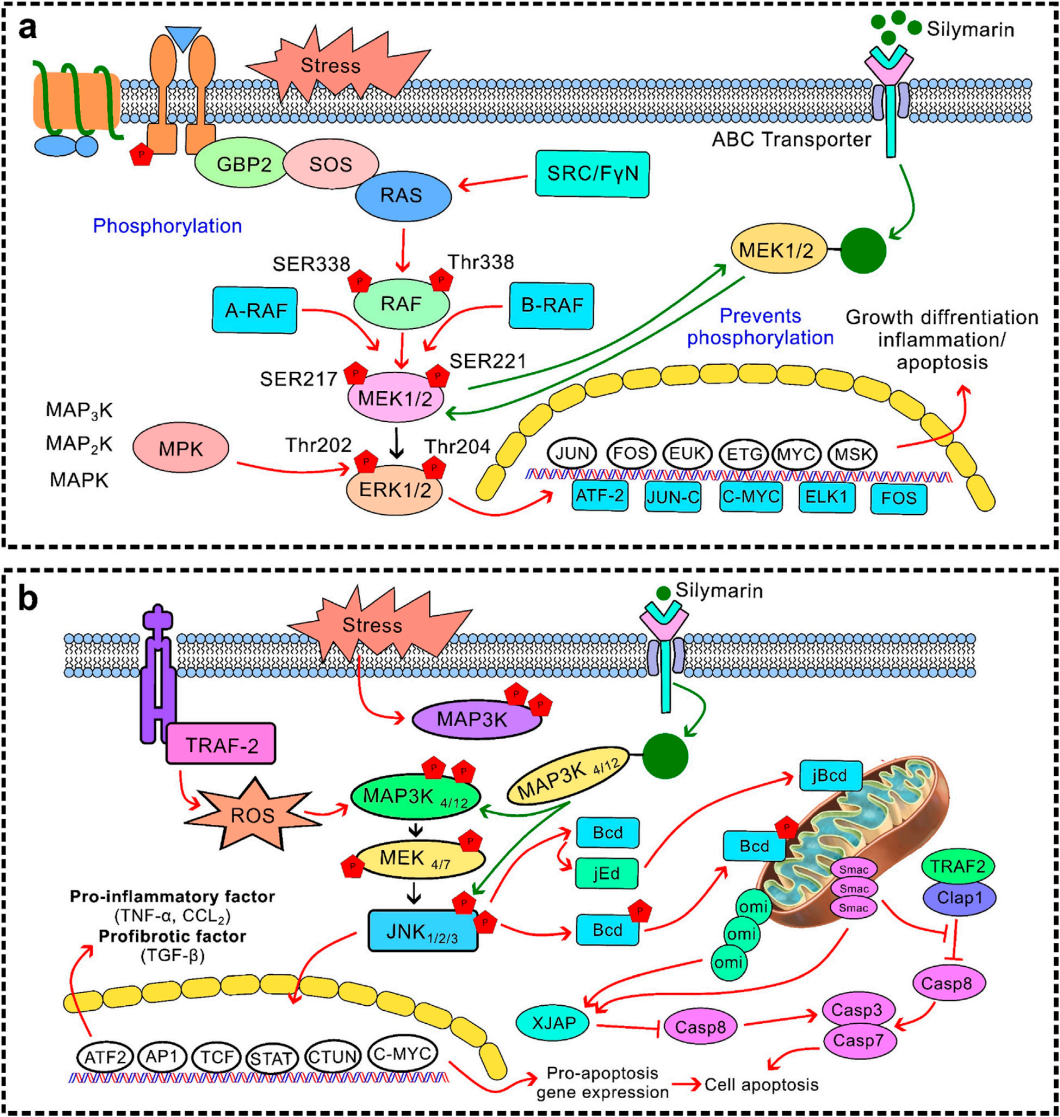
Regulation of MAPK signalling **(a)** Inhibition of the MAPK–GPCR/ERK1/2 pathway: Silymarin inhibits extracellular signal-regulated kinase 1/2 (ERK1/2) phosphorylation, by attenuating cell proliferation and survival signals commonly associated with tumour growth; **(b)** Modulation of the MAPK–JNK pathway: Silymarin stops aberrant Janus kinase activation under stress conditions while simultaneously promoting apoptotic signalling in cancerous cells, leading to the selective induction of apoptosis.

Silymarin have strong anti-inflammatory properties *via* a variety of pathways, chiefly through regulating immune cell activity, cytokine signalling pathways, and inflammasome activation (Lovelace et al., 2015; Esmaeil et al., 2017). Silymarin decreases the expression of pro-inflammatory cytokines, such as TNF-α, IL-1β, IL-6, IL-12, IL-23, CCL4, and CXCL10, by suppressing NF-κB and TLR4/NF-κB signalling (Singh et al., 2021; de Freitas et al., 2024). Additionally, it suppresses JAK/STAT signalling and MAPK pathways (ERK1/2 and p38) (Figures 4a,b), which further reduces the synthesis of inflammatory mediators and cytokines. Furthermore, silymarin inhibits the expression of iNOS, thereby reducing the production of nitric oxide (NO) and subsequently lowering inflammation. It alters the polarisation of macrophages, encouraging a change from pro-inflammatory M1 to anti-inflammatory M2 phenotypes. This results in a decrease in TNF-α, IL-1β, and IL-6 and an increase in IL-4, IL-10, IL-13, and TGF-β. Additionally, it inhibits the JAK/STAT and mTOR pathways, which suppresses T-cell proliferation and pro-inflammatory cytokine release. It also hinders dendritic cell maturation by downregulating the production of IL-12 and co-stimulatory molecules (CD80/CD86), which limits T-cell activation. By altering TXNIP expression, increasing SIRT2 activity, and decreasing mitochondrial ROS and dysfunction, silymarin prevents the activation of the NLRP3 inflammasome. This reduces tissue damage and inflammatory signalling by inhibiting the maturation of IL-1β and IL-18 mediated by caspase-1 (Mai et al., 2023). By focusing on key signalling pathways, controlling immune cell responses, and inhibiting inflammasome activation, silymarin exhibits broad-spectrum anti-inflammatory effects that reduce the production of pro-inflammatory cytokines and maintain tissue homeostasis. These results position silymarin as a multifactorial anti-inflammatory agent that can be effective at both the transcriptional and post-transcriptional levels as well as at the cellular level (Rizwan et al., 2023). The suppression of NF-κB and MAPK signal cascades is the key to this effect, which is why it can be widely used in the treatment of various organ systems.

### 3.3 Organ-specific protective activities

To gain a deeper understanding of the pharmacological actions of silymarin, it is essential to examine the organ-specific mechanisms through which silymarin exerts its effects (Table 2). In addition to its general antioxidant and anti-inflammatory properties, silymarin follows distinct molecular pathways of action, which provide varying protective effects, as discussed below.

**TABLE 2:** Organ-specific molecular mechanisms of silymarin.

Sr. No.	Organ system	Primary protective mechanisms	Key molecular pathways/targets	References
1	Liver	Antioxidant, Anti-inflammatory, Anti-fibrotic, Regenerative	Nrf2, NF-κB, NLRP3 Inflammasome, HSCs, ECM components, RNA Polymerase I, Estradiol Receptor	(Shaker et al., 2010; Gillessen and Schmidt, 2020)
2	Nervous System	Antioxidant, Anti-inflammatory, Anti-apoptotic, Anti-aggregation, Neurotransmitter Modulation	Nrf2, NF-κB, MAPK, p53, Bcl-2/Bax, Caspases, Aβ aggregation, Glutamate release, MAO-B, BDNF	(Borah et al., 2013; Singh et al., 2020)
3	Pancreas	Beta-cell Regeneration, Insulin Sensitization, Antioxidant, Anti-inflammatory, Acinar Cell Protection	Pdx-1, Nkx6.1, ERK1/2, NF-κB, PPARγ, Aldose Reductase, GPX4, Inflammatory Cytokines	Amniattalab et al. (2016)
4	Kidney	Antioxidant, Anti-inflammatory, Anti-fibrotic, Cellular Repair	Nrf2, NF-κB, ROS, Inflammatory Cytokines, TGF-β1, Protein/Nucleic Acid Synthesis	Mohammadi et al. (2024)
5	Cardiovascular System	Antioxidant, Anti-inflammatory, Hypolipidemic, Hypoglycemic, Contractility Modulation	Nrf2, NF-κB, ROS, Inflammatory Cytokines, β1-AR, L-VDCC, Lipid/Glucose Metabolism	Kadoglou et al. (2022)
6	Gastrointestinal Tract	Anti-inflammatory, Anti-cancer	NF-κB, Inflammatory Cytokines, Cell Cycle Regulators, Apoptosis Pathways (p53, Bcl-2/Bax, Caspases), Angiogenesis	Fallah et al. (2021)
7	Bone	Bone Mineralization Stimulation	Undefined specific molecular pathways; experimental evidence suggests direct effect.	Kim et al. (2012)
8	Skin	Photoprotective, Antioxidant, Anti-inflammatory, Anti-carcinogenic	Nrf2, ROS, NF-κB, Inflammatory Cytokines, Cell Cycle Arrest, Apoptosis, CB2R	(Vostálová et al., 2019; Mota et al., 2024)

#### 3.3.1 Hepatoprotective activity

Silymarin has regenerative and hepatoprotective properties. Free radical scavenging, lipid peroxidation prevention, hepatocyte membrane stabilisation, toxin entry limitation, and stimulation of ribosomal RNA synthesis to promote protein formation are some of its mechanisms (Attia et al., 2025). Silymarin reduced hepatomegaly and enhanced glutathione synthesis by increasing cysteine availability, contributing to antioxidant defence. It also decreased collagen accumulation in biliary fibrosis by ∼30% and modestly improved survival in cirrhotic patients. Studies have indicated that silymarin reduces oxidative stress, inflammation (TNF-α, TGF-β1), fibrosis, and markers of hepatic injury (ALT, AST, γ-glutamyl transferase), although vitamin C has occasionally shown better results may be due to vitamin C’s rapid water-soluble antioxidant action and its ability to directly neutralize reactive oxygen species in the aqueous phase (Das and Vasudevan, 2006; Das and Mukherjee, 2012). In individuals with alcoholic hepatopathy, silymarin reduced triglyceride and glucose levels (Lirussi et al., 2002). In patients with cirrhosis, long-term treatment was beneficial in the early stages of the disease, although meta-analyses show mixed results. Overall, silymarin exhibits antioxidant, anti-inflammatory, and anti-fibrotic hepatoprotective properties with a favourable safety profile; however, further studies are necessary to confirm its clinical efficacy.

#### 3.3.2 Cardioprotective activity

According to recent research, silymarin and silibinin show promise as treatments for heart failure, cardiomyopathies, and chemotherapy-induced cardiotoxicity. Silymarin (100 mg/kg) decreased cardiac fibrosis, collagen deposition, and dysfunction in diabetic mice, whereas silibinin used ERK1/2 MAPK signalling to control hypertrophic responses in cardiomyocytes (Meng et al., 2019). Additionally, it increased the expression of the genes TPM1 and MYL2, which are crucial for the heart’s structure and function, indicating potential therapeutic applications in cardiomyopathies (Mazzarotto et al., 2020; Wang et al., 2021). Using antioxidant mechanisms, silymarin demonstrated cardioprotection against chemotherapeutic agents such as doxorubicin, cisplatin, and adriamycin. It also decreased serum biomarkers of myocardial damage (LDH, CK-MB, CPK, and troponin I) and lessened histological damage (El-Shitany et al., 2008; El-Awady et al., 2011; Razavi and Karimi, 2016). Despite its limitations, clinical evidence suggests positive outcomes for patients with metabolic disorders, dyslipidaemia, and type 2 diabetes mellitus (T2DM). Improved glycaemic control, lipid profiles, insulin sensitivity, and decreased inflammation are reported by meta-analyses and randomised controlled trials (Mohammadi et al., 2019; Xiao et al., 2020). Although study heterogeneity and small sample sizes restrict definitive conclusions, positive outcomes have also been observed in patients with diabetic nephropathy, alcoholic liver disease, coronary artery bypass graft patients, and non-alcoholic fatty liver disease (Altaei, 2012; Voroneanu et al., 2017; Federico et al., 2019). Overall, silymarin shows promise as a cardioprotective agent through antioxidant, anti-fibrotic, and metabolic regulatory actions, warranting further clinical validation.

#### 3.3.3 Renoprotective activity

The oxidative and inflammatory burden to which the kidney is exposed is high due to its role in xenobiotic detoxification. The renoprotective effect of silymarin is achieved through inhibitory effects on oxidative stress, maintenance of renal tubular integrity, and reduction of pro-inflammatory cytokines. Silymarin’s protective function in renal diseases, specifically diabetic nephropathy, drug-induced nephrotoxicity, and end-stage renal disease (ESRD), has been studied. According to preclinical research, silymarin reduces renal damage by having anti-inflammatory, anti-fibrotic, and antioxidant properties. Silymarin supplementation, either by itself or in conjunction with vitamin E, decreased plasma malondialdehyde (MDA), enhanced haemoglobin levels, and improved red blood cell counts in ESRD patients receiving haemodialysis or peritoneal dialysis (Georgiev et al., 2023). Silymarin and renin-angiotensin system inhibitors did not significantly improve overall survival or renal progression in type 2 diabetic nephropathy, according to randomised controlled trials; however, benefits were noted in subgroups with higher proteinuria or lower estimated glomerular filtration rate (eGFR) (Fallahzadeh et al., 2012). Additionally, silymarin was shown in animal studies to have renoprotective effects against nephrotoxicity caused by cisplatin, adriamycin, and other chemotherapeutic agents, as indicated by decreased blood urea nitrogen, serum creatinine, and oxidative stress markers. Overall, silymarin shows potential as a supportive agent in renal dysfunction through its antioxidant and anti-fibrotic properties. However, the limited number of clinical trials, small sample sizes, and study heterogeneity highlight the need for further well-designed studies to confirm its therapeutic role.

#### 3.3.4 Dermato-protective activity

Due to their anti-inflammatory and antioxidant properties, silymarin has potent photoprotective effects (Anthony and Saleh, 2013; Pittayapruek et al., 2016). Photoaging is caused by the induction of ROS, activation of MMPs, and degradation of ECM proteins brought on by UVA and UVB irradiation. Fenton chemistry and MMP activity are inhibited by silymarin and its flavonolignans because they function as metal chelators and radical scavengers (Vimalraj et al., 2018). Although its instability and phototoxicity restrict its use, Silymarin efficiently inhibited collagenase and elastase, with DHSB exhibiting the highest elastase inhibition and radical scavenging activity (Rajnochová Svobodová et al., 2016). In addition, Silymarin and flavonolignans directly absorb UV photons, conferring measurable sun protection factors (SPF, UVA-PF), with SB most effective in the UVB range and DHSB in the UVA range (Couteau et al., 2012; Rajnochová Svobodová et al., 2016). Overall, the multicomponent silymarin extract provides more stable and broader anti-photoaging protection than individual flavonolignans, making it a cost-effective candidate for dermatological formulations. In skin conditions such as psoriasis and acne, silymarin downregulates keratinocyte hyperproliferation and NF-κB signalling, which provides relief from symptoms (Vostálová et al., 2019; Behera et al., 2025; Sutar and Shukla, 2025). Accordingly, Silymarin offers dermatoprotection with dual roles, prophylactic and therapeutic.

#### 3.3.5 Anticancer activity

**FIGURE 5:**
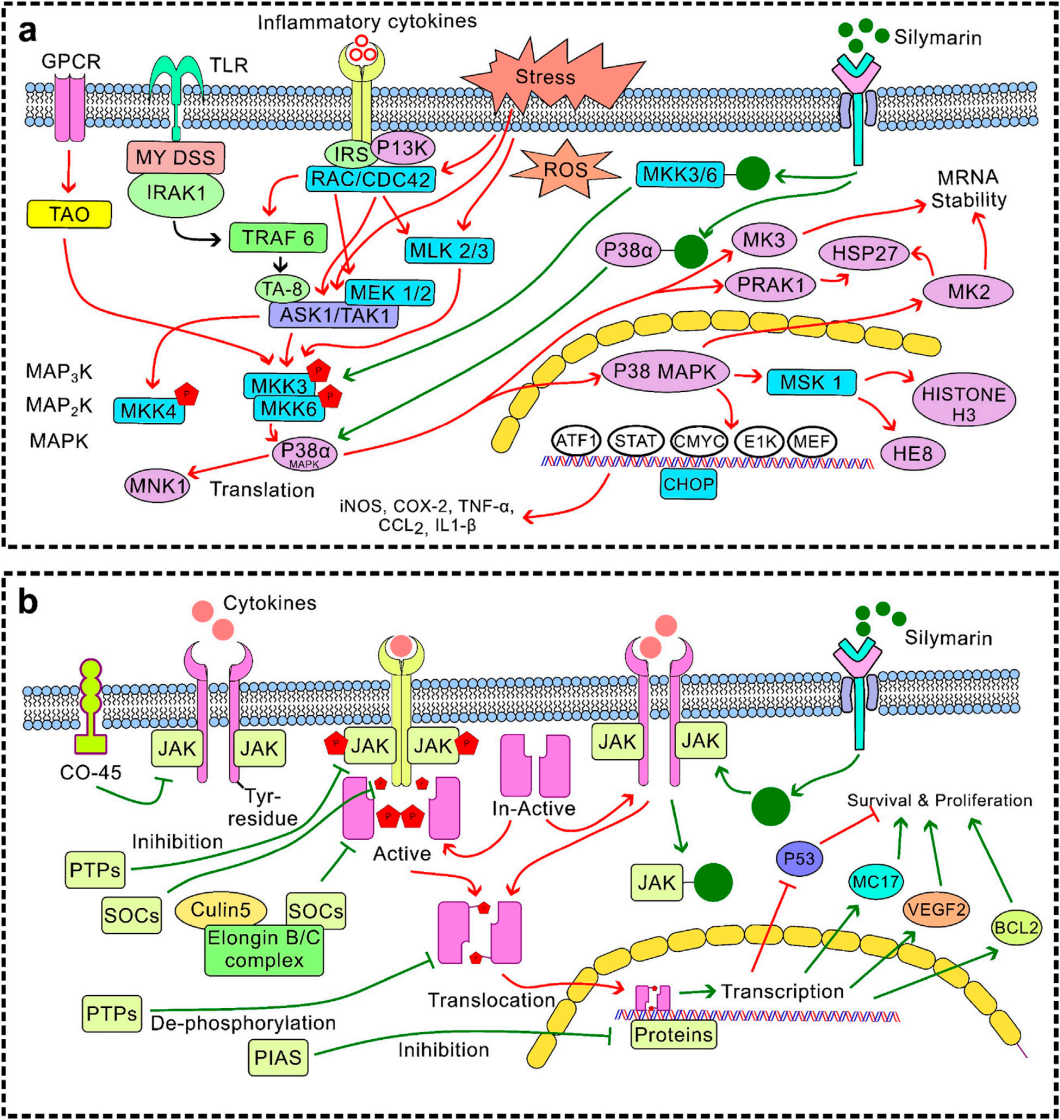
Stress and survival signalling pathway **(a)** Inhibition of the MAPK–p38 pathway: Silymarin reduces phosphorylation of p38 MAPK, by attenuating inflammatory responses, while in cancer cells it can shift the balance toward apoptosis and growth suppression; **(b)** JAK/STAT suppression: Silymarin inhibits Janus kinase–mediated activation and nuclear translocation of signal transducer and activator of transcription (STAT) proteins, leading to decreased expression of genes involved in proliferation, angiogenesis, and survival.

**FIGURE 6:**
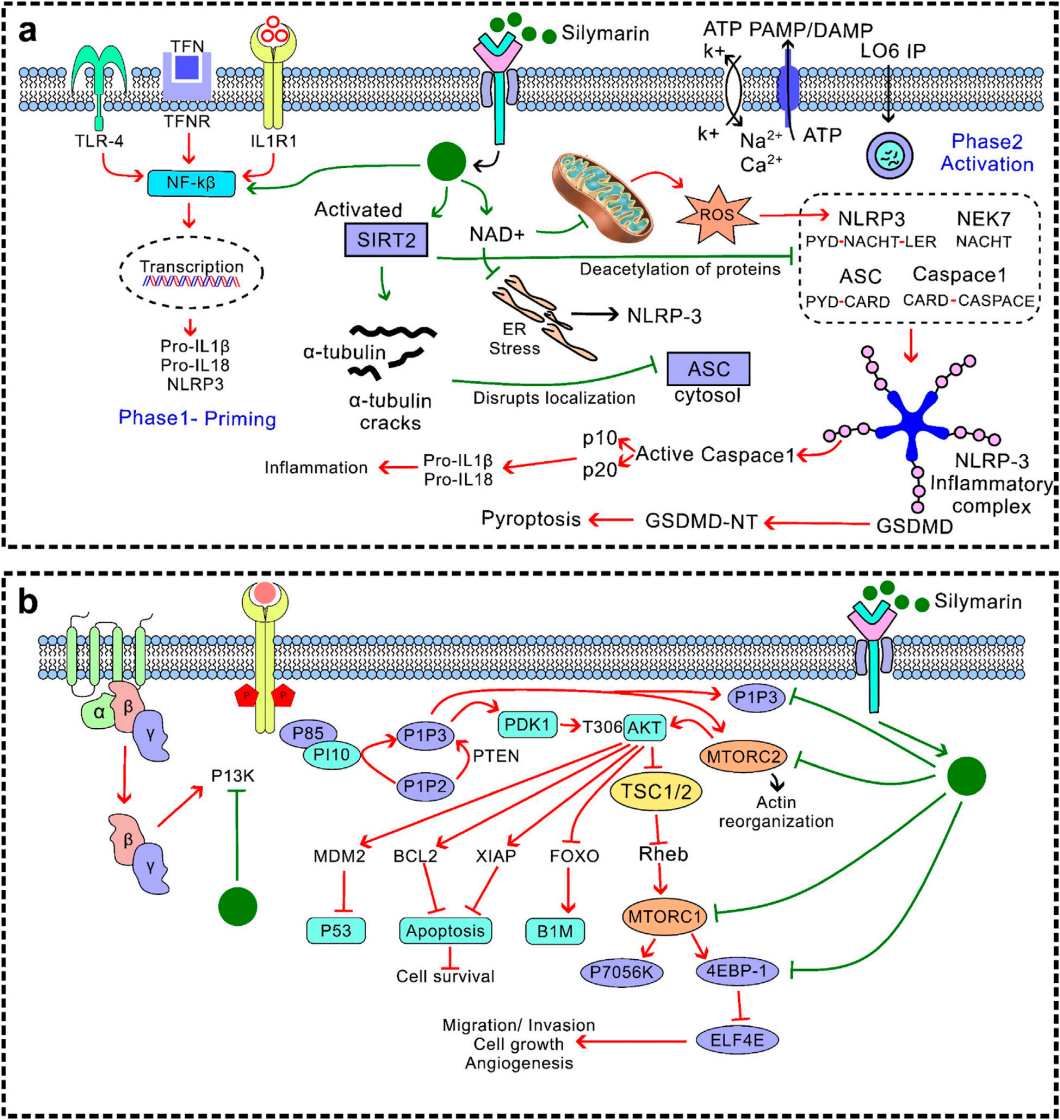
Anti-inflammatory and antiproliferative signalling **(a)** Inhibition of the NLRP3: Silymarin suppresses NLRP3 activation and subsequent assembly of the inflammasome complex, thereby reducing caspase-1 activation and the maturation of pro-inflammatory cytokines such as IL-1β and IL-18. This leads to attenuation of inflammation-driven tissue injury; **(b)** PI3K/Akt/mTOR inhibition: Silymarin interferes with phosphatidylinositol 3-kinase (PI3K) activation and downstream phosphorylation of Akt and mTOR, resulting in decreased cell growth, survival, and angiogenesis.

Silymarin has attracted interest due to its multi-targeting capacity to modulate cancer markers (Figures 5, 6) and mitigate the toxicities associated with chemotherapy in both *in vitro* and *in vivo* models. However, its low solubility, stability, and bioavailability limit its clinical translation. Nano delivery systems have been created to address these issues by preventing silymarin from degrading, enhancing its pharmacokinetics and solubility, facilitating controlled and targeted release, and lowering systemic toxicity. In comparison to free silymarin, nanoformulations offer sustained release, which reduces the possibility of adverse effects (Wang et al., 2023). It leads to cell cycle arrest at the G1/S and G2/M phases through the decreased activity of cyclin D1 and CDK4, as well as the upregulation of p21 and p27. It also induced apoptosis through the mitochondrial pathway, increasing Bax/Bcl-2 ratio and activating caspase-9 and caspase-3. *In vitro* studies in breast cancer models showed that the tumour volume decreased by 55% and indices of apoptosis improved upon silibinin treatment. The preparation techniques, particle size, surface charge, internalisation mechanisms, intracellular metabolism, biodistribution, and carrier materials are among the formulation parameters that affect the therapeutic efficacy of silymarin-loaded nanoparticles. Limitations still exist, despite promising preclinical data, most notably the scarcity of clinical trials and our incomplete understanding of potential toxicities in humans. Therefore, to confirm safety, improve formulations, and demonstrate the efficacy of silymarin nanoformulations as potent anticancer treatments, carefully planned clinical trials are necessary. It has been demonstrated to possess a wide range of anti-cancer properties, including anti-proliferative, pro-apoptotic, anti-angiogenic, and anti-metastatic effects.

#### 3.3.6 Neuroprotective activity

Silymarin has been consistently shown to have neuroprotective effects through estrogen receptor-mediated, antioxidant, and anti-inflammatory pathways. It shields neurones from oxidative damage and nitrosative stress, primarily by lowering lipid and protein oxidation, increasing enzymatic and non-enzymatic antioxidant markers, and reviving mitochondrial activity (Moore et al., 2005; Lu et al., 2009; Baluchnejadmojarad et al., 2010). Silymarin improved acetylcholinesterase function, decreased malondialdehyde, inhibited myeloperoxidase activity, and restored glutathione levels in models of sepsis, encephalopathy, and manganese-induced neurotoxicity. It reduced neuronal damage in aged rodent brains by preventing the production of oxygen/peroxyl radicals and protein oxidation (Vengerovskii et al., 2007; Toklu et al., 2008; Chtourou et al., 2010). It improved Aβ clearance, prevented Aβ oligomerisation and fibril aggregation *in vitro* and *in vivo*, and reduced Aβ-induced cognitive impairment by lowering lipid peroxidation and restoring glutathione in the hippocampus in Alzheimer’s disease models (Murata et al., 2010; Yin et al., 2011). Additionally, it suppressed inflammatory mediators, such as TNF-α and iNOS, and reduced nitrotyrosine levels in the hippocampus and amygdala, thereby attenuating Aβ-induced nitrosative stress. Interestingly, this protective effect was observed without affecting the activity of β-secretase (BACE), a crucial enzyme in the production of Aβ (Hong et al., 2000; Murata et al., 2010). Additionally, silymarin alters glial responses. It suppressed iNOS and NF-κB activation in glial cells, prevented astroglial and microglial oxidative injury in *ex vivo* systems, and inhibited microglial activation in lipopolysaccharide (LPS)–induced Parkinson’s disease (PD) models (Wang et al., 2002). Furthermore, Silybinin, a key constituent, increased anti-inflammatory markers and decreased chemically induced inflammation by activating the Akt/mTOR pathway. The estrogen receptor-β (ER-β), which is primarily expressed in the hippocampus and cortical regions, is another significant mechanism. While its blockade leads to neurodegeneration, ER-β activation is linked to neuroprotection in learning and memory (Hill and Boon, 2009; Hughes et al., 2009). By regulating ER-β, silymarin reduced 6-OHDA-induced dopaminergic loss and motor dysfunction in PD models, indicating its estrogen-like activity and capacity to bind/activate ER-β. Despite silymarin’s strong neuroprotective potential, significant gaps in our understanding remain. It is unclear exactly how Aβ fibrillization is inhibited in the absence of BACE modulation. Its possible function in inhibiting α-synuclein aggregation and Lewy body formation in Parkinson’s disease is still unknown. Furthermore, the blood–brain barrier permeability and bioavailability of Silymarin are still unknown, necessitating further research into its pharmacokinetics and mechanistic foundations.

#### 3.3.7 Interaction with mitochondrial and hepatic receptors

Silymarin’s therapeutic efficacy is regulated by its interactions with mitochondrial and hepatic receptor systems, which together control its bioactivation and intracellular signalling responses. Within hepatocytes, silymarin shows a high affinity for membrane-bound and nuclear receptors, including PPAR-α and CAR/PXR, by enhancing the expression of hepatic detoxification enzymes and improving xenobiotic clearance (Yu et al., 2025). Its mitochondrial interaction occurs primarily through the stabilisation of mitochondrial membranes and modulation of oxidative phosphorylation, preserving ATP synthesis while preventing cytochrome c leakage and subsequent apoptosis (García-Muñoz et al., 2024). These synergise with receptor-mediated activation of Nrf2 (Figure 3a) and inhibition of NF-κB (Figure 3b), promoting antioxidant defence. Moreover, by attenuating MAPK–ERK and PI3K/Akt/mTOR signalling cascades (Figures 4–6), silymarin reduces oxidative stress, thereby prolonging hepatocellular survival and optimising pharmacokinetic stability through improved metabolic resilience. In summary, these mitochondrial–receptor interactions underpin silymarin’s hepatoprotective efficacy and explain its favourable therapeutic potential, despite its limited systemic bioavailability.

## 4 Pharmacokinetics profile of Silymarin

Silymarin, a combination of flavonoids, has been the subject of numerous studies regarding its hepatoprotective, antioxidant, anti-inflammatory, and immunomodulatory properties (Rašković et al., 2011; Abbas et al., 2020; Yanaşoğlu et al., 2022). Its clinical efficacy is largely limited by its pharmacokinetics due to low aqueous solubility, poor intestinal permeability and high first-pass catabolism in the intestine (Song I. S. et al., 2021; Xu et al., 2022). These result in highly impaired oral bioavailability, considerable exposure variability, and significant variability of therapeutic performance in diminished practice (Seo S. R. et al., 2024; Chang et al., 2025).

### 4.1 Absorption

Silymarin exhibits low aqueous solubility and poor permeability, resulting in low oral bioavailability (approximately 23%–47%). Active plasma concentrations of flavonolignans range from 50 to 300 ng/mL, with a T_max_ of 6–8 h, suggesting low and inefficient uptake of flavonolignans. This is due to its low oral bioavailability, which, combined with its swift metabolism, accounts in large part for why *in vitro* results do not always translate well clinically (Xu et al., 2022).

### 4.2 Distribution

After absorption, silymarin is distributed to all tissues quickly and extensively, but concentrates mainly in metabolically active ones. Highest levels have been observed in liver, lungs, stomach, skin, prostate and pancreas within an hour of administration in various animal models (Marhol et al., 2015). Liver shows the highest absorption, with biliary concentration of silibinin being up to 100-fold as compared to plasma levels, since active concentrating mechanisms in the liver magnify circulating levels in hepatocytes (Sun Y. et al., 2025). This hepatotropic distribution gives a robust mechanistic rationale as to why silymarin can be used in the clinic to treat liver disorders (Rašković et al., 2011).

The protein binding is moderate, with a range of 46%–70.3% in preclinical studies; compared with lower or no protein binding that can prevent tissue penetration, as well as the lower protein binding and higher free fraction of other analogues, these valences have the same effect (Marhol et al., 2015; Li et al., 2019b; Chi et al., 2020; Chang et al., 2025).

### 4.3 Metabolism

The clearance of silymarin involves considerable first-pass, which is largely by phase II conjugation. This metabolism is done by UDP-glucuronosyltransferase (UGT) isoforms (mainly UGT1A1, 1A6, 1A7, 1A9, 2B7, and 2B15), which account for about 55% of derivatisation and 28% by sulfation (Vrba et al., 2020). The stereoselective glucuronidation of silybin B and silybin A at C-20 and C-7 and C-20, respectively, has been confirmed, and the roles that C-20 and C-7 glucuronidation play in the *in vivo* disposition of silybins remain to be elucidated. Sulfation, catalysed by sulfotransferases (SULTs), also plays an important role in total clearance, where both plasma and urinary analysis have revealed multiple conjugated forms (glucuronide, sulfate, mixed) (Vrba et al., 2020). Phase I metabolism is involved to a lesser extent, with about 17% of the total clearance, vis-a-vis demethylation of the form, CYP2C8 acting as a catalyst to O-demethylation to produce hydroxylated derivatives (Brantley et al., 2013; Sun Y. et al., 2025).

### 4.4 Excretion

Silymarin is excreted through the biliary route mainly as conjugated metabolites. Less than 3% of the administered dose is excreted in the urine as the unchanged drug, and renal clearance constitutes no more than 1%–2% of the administered dose over a 24-h period. The very large biliary-to-blood concentration ratio (AUC_bile_/AUC_blood_ = 30 ± 9.4) demonstrates that the active hepatobiliary route is the primary route of elimination. Additional delay in systemic availability occurs due to the enterohepatic circulation of glucuronidated compounds, as biliary excretors become exposed to gastrointestinal bacterial hydrolysis and are subsequently reabsorbed. This recycling process, however, mainly comprises conjugates whose drug activity is low (Pferschy-Wenzig et al., 2023).

## 5 Strategies to enhance bioavailability

In recent years, many different platforms of delivery have been used in an attempt to overcome the solubility issue of silymarin, metabolic breakdown processes, and absorption into rich lipid-like states of the intestinal lining (Chang et al., 2025). This section presents a critical review of the key delivery strategies proposed for silymarin delivery at the site of action, along with the major systems involved in enhancing bioavailability (Table 3).

**TABLE 3:** Major delivery systems for improving silymarin bioavailability.

Delivery platform	Structural features	Mechanistic basis of bioavailability enhancement	References
Micelles	Amphiphilic surfactant assemblies (composed of Tween 80, PEG-DSPE, etc.), forming nanosized colloidal structures	Solubilization of hydrophobic flavonolignans; improved intestinal permeability via surfactant-mediated membrane fluidisation	Garg et al. (2022)
Liposomes	Lipidic bilayer vesicles encapsulating molecules	Protection from enzymatic degradation; lymphatic uptake; controlled release via phospholipid interaction with bile salts	Wu et al. (2025)
Nanoemulsions	Oil-in-water emulsions (droplet size <200 nm) stabilised with surfactant	Increased interfacial surface for absorption; improved solubilization	(Parveen et al., 2015; Mohylyuk et al., 2021)
Solid Lipid Nanoparticles	Solid lipid core (e.g., glyceryl behenate, stearic acid) with surfactant coating	Controlled release via lipid crystallinity; protection from hydrolysis	Eroglu et al. (2024)
Phytosomes	Complexes of silymarin with phosphatidylcholine	Enhanced lipophilicity and membrane permeability via complex formation	Maryana et al. (2016)
Polymeric Nanoparticles	Biodegradable polymers (PLGA, chitosan, PEGylated systems) entrapping silymarin	Protection from enzymatic degradation, controlled release	Mishra et al. (2023)

### 5.1 Self-emulsifying drug delivery systems (SMEDDS)

SMEDDS are an isotropic blend of oils, surfactants and cosolvents that form fine oil-in-water emulsions when in contact with gastrointestinal liquids. In a few studies, a large improvement in solubility along with oral bioavailability of silymarin has been observed after forming SMEDDS. As an example, Clinical studies of silymarin SMEDDS demonstrated a 3.6–4.1-fold increase in AUC and a 4-fold increase in C_max_ compared to conventional extracts, with t_1_/_2_ extending from 2.5 to 6.1 h (Sornsuvit et al., 2018). Specifically, a 140 mg SMEDDS formulation yielded a mean C_max_ of 812.43 ng/mL at 0.8 h and ${\text{AUC}}_{0-\infty }$ of 676.98 ng·h/mL. Micellar formulations also enhanced bioavailability, showing a 4.2-fold increase in C_max_ and improved dissolution rates in human trials (Chang et al., 2025).

In micellar systems, notably those with polymeric surfactants such as Pluronic F127 or d-alpha-tocopherol polyethylene glycol succinate (TPGS) have been shown to have both synergistic properties of solubilization and inhibition of P-glycoprotein (P-gp) efflux. TPGSs formulated encapsulating silymarin, introducing particle sizes of less than 100 nm, encapsulation efficiency of more than 90%, and ∼5.2-fold improvement in the bioavailability of the drugs accompanying oral administration in animal models (Piazzini et al., 2019; Song I.-S. et al., 2021). Intestinal permeability was also increased by TPGS efflux inhibition. These results demonstrate that SMEDDS and micelles are viable delivery vehicles, but surfactant-related toxicity should be considered.

### 5.2 Nanocrystals

Nanocrystals reflect a purely nanoparticulated drug particle approach, with no solubilising excipient effects, thereby retaining high drug loading. When the particle size is reduced to nanometric units, the surface area and the dissolution rate increase.

Seo et al. demonstrated the preparation of silymarin HM40 nanocrystals using a modified wet-milling method and their subsequent good solubility (mean particle size of ∼645 nm and increased solubility by 6-fold compared with crude silymarin) (Seo S. et al., 2024). The *in vivo* human studies revealed a 1.5-fold increase in AUC. Another ingenious system is the approach to lipid nanoliquid crystal, where amphiphilic lipids are combined to achieve bicontinuous cubic phases. These systems demonstrated a good drug loading capacity, stability, and a ∼3-fold increase in oral bioavailability in rat models, outperforming conventional lipid carriers.

### 5.3 Solid dispersions and polymer-based systems

Solid dispersions disperse the drug in hydrophilic carriers on the molecular level, which enhances wettability, amorphisation and the rate of dissolution. Silymarin is combined with various polymeric systems, including PVP, HPMC, PEG, and Soluplus.

To achieve partial amorphisation of silymarin, solid dispersions are prepared using water-soluble polymers (PVP, HPMC), which ensures better wettability and faster dissolution (Song I.-S. et al., 2021). Configurations of 2.1- and 2.7-fold increased oral bioavailability in animal models were achieved using binary or ternary solid dispersions (Alkathiri et al., 2024). Mechanistically, the effects of polymer drug interactions are the maintenance of supersaturation, prevention of precipitation and enhancement of solubility-based drug absorption. The amorphous drug is further stabilised as advanced formulations, including spray-dried dispersions, are used.

### 5.4 Phytosomes/phospholipid complexes

Phytosomes are complexes of plant bioactives with phospholipids (usually phosphatidylcholine), typically of the amphiphilic type with augmented solubility and penetration properties. The phytosomes approach is one of the best clinically verified methods, with various commercial products (e.g., Siliphos) already available worldwide, which also indicates its translational advantage over other experimental models.

Méndez-Sánchez et al. found that a silybin–phosphatidylcholine complex in oily-medium soft-gel capsules provided superior bioavailability compared to conventional silymarin tablets (Méndez-Sánchez et al., 2019). In animal models, complexes of silymarin and phospholipids had an increased oral bioavailability by 4-fold and greater hepatoprotective efficacy than unformulated silymarin (Shriram et al., 2022).

### 5.5 Cyclodextrin inclusion complexes

Cyclodextrins (CDs) enhance silymarin solubility and dissolution, with HP-β-CD increasing solubility ∼20-fold and oral bioavailability ∼2.0–2.1-fold in rats (Imam et al., 2023). They protect against enzymatic degradation and improve wetability, but limited complexation and high-dose toxicity remain, partly addressed by modified derivatives.

### 5.6 Lipid nanoparticles

Lipid-based nanoparticles such as solid lipid nanoparticles (SLNs) and NLCs have the potential to protect against enzymatic degradation by lipases and release in a controlled manner and *via* a lymphatic path of delivery.

NLC-based silymarin delivery (∼150–200 nm) has exhibited 8-fold enhanced oral bioavailability, longer T_1/2_, and enhanced hepatoprotective profile in acetaminophen-induced liver damage models in preclinical studies (Iqbal et al., 2018). Nanoemulsions also offer a small droplet size, which enhances dissolution. In a study by Parveen et al., a silymarin nanoemulsion was developed using Tween 80 as a surfactant, resulting in a droplet size of 41.22 ± 0.00314 nm. This formulation demonstrated a 4-fold increase in oral bioavailability compared to a silymarin drug suspension in rats (Parveen et al., 2015).

### 5.7 Liposomes and vesicular carriers

Liposomes provide the benefits of a biocompatible phospholipid bilayer carrier that can trap hydrophilic and hydrophobic compounds. Liposomes loaded with silymarin to an extent of ∼120–135 nm had 34-fold higher oral bioavailability as compared to free silymarin, with an increased hepatoprotective activity in CCl 4 and copper-induced liver injury models, with a significant decrease in ALT, AST, and indices of oxidative stress (Maryam et al., 2023; Wu et al., 2025). Mechanistically, absorption occurs through transcellular passage and endocytosis into the enterocytes. PEGylation or bile salt surface modification extends the transit time in the intestine and enhances stability. The effect on radical scavenging activity under UV irradiation, as well as lyophilisation, is also presented by liposomal encapsulation (Karkad et al., 2025).

Newer vesicular systems, such as transferosomes and niosomes, have also been tested. Researchers found that silymarin-loaded transferosomes showed improved penetration of the drugs into the skin and absorption into the body, which could be used as a transdermal delivery (Abdallah et al., 2022).

### 5.8 Metal–organic frameworks (MOFs) and novel hybrids

New studies have explored the MOFs and inorganic-organic hybrid systems to encapsulate silymarin. They are porous crystalline materials with a large surface area that can be used to tune the pore environment. CME@ZIF-8 MOFs are hybrid nanoplatforms, which display high drug loading, modulated release, and mechanistic synergy in oxidative stress mitigation. MOFs loaded with silymarin showed a 4.2-fold increase in plasma levels *in vivo*, and substantially prevented chemically induced hepatic injury (Yu et al., 2023).

### 5.9 Permeation enhancers and efflux inhibitors

In addition to the use of carriers, co-administration strategies using bioenhancers have also been extensively investigated. Piperine, a natural alkaloid of black pepper, inhibits the glucuronidation process and increases intestinal permeability. In the study, Concomitant administration of silybinin (silymarin) and piperine was demonstrated to improve the bioavailability by almost 146%–181% in rat hepatocytes (Bi et al., 2019).

The TPGS is also demonstrated to increase P-gp inhibition and reduce P-gp efflux, thereby increasing intestinal transport of silymarin, in addition to its solubilising activity. These methods emphasise the prospects of combining pharmacological and formulation-related techniques for the greatest effect.

### 5.10 Co-formulation and organ-targeted strategies

The therapeutic potential of silymarin is limited by its poor oral bioavailability, primarily due to low aqueous solubility and active efflux mechanisms mediated by intestinal transporters, such as BCRP and MRP2. Co-formulation, specifically the incorporation of synergistic compounds, provides a critical solution by overcoming these kinetic barriers and enabling multi-organ targeting. Pharmacological efflux pump inhibitors directly enhance absorption; co-administration with piperine, an inhibitor of BCRP and MRP2, showed a 60% increase in the C_max_ of total silybin B, while the flavone baicalein similarly boosted the AUC and C_max_ of silybin (Xu et al., 2018). Combining efflux inhibition with advanced delivery systems resulted in even greater success; solid dispersion formulations utilising the excipient D-α-Tocopheryl polyethylene glycol 1000 succinate (TPGS) leverage its P-gp inhibitory properties alongside solubilization, resulting in a 4.0-fold increase in plasma C_max_ (Mohylyuk et al., 2021). This enhanced systemic exposure facilitates multi-organ efficacy, ensuring metabolites reach critical extra-hepatic tissues, such as the kidneys, adrenals, and bone marrow, and allows for the development of specialised nanocarriers to cross the blood-brain barrier for neuroprotective applications.

## 6 Clinical evidence

The clinical status of silymarin in liver disease is limited. In literature, placebo-controlled clinical trials involving 1,209 participants, treatment with standardised silymarin formulations, such as Legalon or Silipide, showed no statistically significant improvement in mortality, histological findings, or major biochemical markers of hepatic function compared with the placebo (ClinicalTrials.gov, 2024). The pooled odds ratio for mortality was 0.8 (95% CI, 0.5–1.5; P = 0.6), showing no survival benefit. The observed biochemical effects were minor, specifically a 9 IU/L reduction in alanine aminotransferase (ALT) among patients with chronic liver disease. Histological improvement on biopsy was inconsistently reported. Importantly, silymarin was well tolerated, with adverse event rates of 2%–10%, which were indistinguishable from those of the placebo; reported effects were mainly mild gastrointestinal or dermatologic symptoms. Despite using a novel formulation (e.g., *Silipide*, a silybin-phosphatidylcholine complex), the analysis highlighted pharmacokinetic variability and limited systemic exposure, which may underlie the modest therapeutic efficacy observed in humans (Jacobs et al., 2002; García-Muñoz et al., 2024; ClinicalTrials.gov, 2024). Collectively, these clinical findings underscore that while silymarin appears safe, its therapeutic efficacy, optimal dosing, and pharmacokinetic behaviour in human liver disease remain inadequately defined, emphasising the need for larger, well-controlled clinical trials along with a focus on its formulation development.

## 7 Limitations of silymarin studies and PAINS considerations

Silymarin and other polyphenolic compounds, despite extensive research on their potential pharmacological effects, should be interpreted with caution due to the chemical nature of polyphenolic compounds, which can act as pan-assay interference compounds (PAINS). PAINS are known to generate false-positive or artefactual findings in biochemical and cellular assays due to non-specific interactions that are not related to true pharmacological effects (Moreira et al., 2021). The silymarin flavonolignans (e.g., silybin, isosilybin, silychristin) each have a number of hydroxyl and phenolic groups in their structures. In theory, these functionalities can lead to redox cycling, metal chelation, and covalent modification of protein residues. Such activities, if permitted to occur during *in vitro* or *in silico* assays, will likely lead to misleading results (Baell and Nissink, 2018; Moreira et al., 2021). Additionally, silymarin’s natural fluorescence, tendency to aggregate at high concentrations, and reactivity with assay reagents can confound the interpretation of standard colourimetric or fluorescence-based assays employed to assess enzyme inhibition, ROS scavenging, or receptor binding (Magalhães et al., 2021). These physicochemical properties complicate the ability to differentiate between *bona fide* target-specific effects and nonspecific assay artefacts. For example, reports of enzyme inhibition or modulation of signalling by silymarin derivatives in *in vitro* systems might partly reflect nonspecific adsorption or redox interference, rather than a real biochemical effect. As such, while *in vitro* investigations provide important mechanistic insights, their results cannot be directly generalised to pharmacological or clinical implications without rigorous validation. This highlights the importance of orthogonal assays, proper positive and negative controls, and well-characterised reference compounds in demonstrating specificity. Additionally, *in vivo* research and carefully designed clinical trials continue to be crucial for demonstrating the biological significance of silymarin’s effects within the physiological context. Acknowledging and controlling for PAINS-related artefacts elevates the credibility of future silymarin research and aids in the rational development of silymarin as a phytopharmaceutical. It is essential to critically examine these limitations when attempting to derive clinical significance from *in vitro* discoveries.

## 8 Future perspectives and conclusion

Clinical applications of silymarin will depend upon the formulation in which we are formulating silymarin to improve its bioavailability. From literature, SMEDDS found a modest 3.6–4.1-fold increase in oral bioavailability, whereas nanocrystals provided 1.5-fold improvements in absorption (Sornsuvit et al., 2018; Seo S. et al., 2024). Metal-organic frameworks and other systems, such as CME@ZIF-8, have also been explored and have shown an improved hepatoprotective effect; however, their scale-up, regulatory approval, and toxicological examination remain major translational limit. This suggests that toxicological and biodistribution studies should be conducted before clinical deployment.

Since liver disease, neurodegenerative diseases and cancer are antagonised by multiple factors, their combination therapy with silymarin and other medications has synergistic potential. Pilot studies anecdotally report substantial benefits in ALT/AST reduction using vitamin E and antioxidant complexes; however, these trials are limited by small sample sizes and short-term follow-ups. In oncology, combinations involving curcumin or vincristine have demonstrated preclinical efficacy against NF-κB-mediated resistance; however, clinical validation has not been conducted. We strongly need robust randomised studies that use either histological or survival as an endpoint.

Silymarin has a rich history of use and strong preclinical data supporting its antioxidative, anti-inflammatory, hepatoprotective, neuroprotective, anti-fibrillary, anticarcinogenic, and immunomodulatory characteristics. However, it has low water solubility, low gastric absorption, and a severe first-pass effect, resulting in poor bioavailability and unpredictable efficacy. Recent advances in formulation science have aimed to overcome these shortcomings. Liposomes, polymeric nanoparticles, solid lipid nanoparticles, phytosomes, and self-emulsifying systems significantly facilitate solubilization, absorption, and targeting to selected tissues, thereby enhancing pharmacological performance. These platforms overcome not only pharmacokinetic obstacles but also enable tissue-specific delivery, resulting in greater efficacy across various patient populations.

Future translation of silymarin-based therapeutics into clinical practice faces several regulatory challenges. Important among these are inter-batch variability, lack of pharmacopoeial standards, and inadequate stability and reproducibility data across different formulations. This must be addressed to ensure consistency and clinical reliability. In the future, various approaches like green synthesis, standardised extract profiling, and AI-assisted optimisation of delivery systems could accelerate silymarin’s clinical translation.

## Glossary

**Rel A**: v-Rel Avian Reticuloendotheliosis Viral Oncogene Homolog A
**p50**: NF-κB pro subunit
**COX-2**: Cyclooxygenase-2
**iNOS**: Inducible Nitric Oxide Synthase
**ICAM-1**: Intercellular Adhesion Molecule-1
**MCP-1**: Monocyte Chemoattractant Protein-1
**MP-1**: MEK Partner 1
**IP-10**: Interferon gamma-induced protein 10
**TNF-α**: Tumour Necrosis Factor-α
**IL-1β**: Interleukin 1 beta
**IL-16**: Interleukin 16
**GPCR**: G-Protein Coupled Receptor
**GWF**: Glycogen 6-phosphate dehydrogenase growth factor
**GBR2**: GABA Receptor Subunit beta-2
**RAS**: Rat Sarcoma (family of small GTPase protein)
**RAF**: Rapidly Accelerated Fibrosarcoma
**A-RAF**: A-Rapidly Accelerated Fibrosarcoma
**B-RAF**: B-Rapidly Accelerated Fibrosarcoma
**MPK**: Mitogen Activated Protein Kinase
**MEK1/2**: MAPK/ERK kinase 1 and 2
**ERK1/2**: Extracellular Signal-Regulated Kinase 1 and 2
**MAP3K**: Mitogen activated protein kinase
**MAP2K**: Mitogen activated protein kinase
**MAPK**: Mitogen Activated Protein Kinase
**TRAF-2**: TNF Receptor associated factor-2
**MAP3K4**: Mitogen-Activated Protein Kinase 3-4
**MAP3K12**: Mitogen-Activated Protein Kinase 3-12
**MKK**: MAPK Kinase
**MEKK1/4/7**: MAPK/ERK kinase 1, 4 and 7
**JNK1/2/3**: c-Jun N-terminal kinase 1, 2 and 3
**Bcl**: B-cell lymphoma
**J Bcl**: variant of Bcl
**omi**: omi/HtrA2 (Apoptosis protease)
**Smac**: Second mitochondria derived activator of caspases
**clp-1**: Claspin 1
**casp8**: Caspase 8
**casp7**: Caspase 7
**casp3**: Caspase 3
**ATF-2**: Activating Transcription factor 2
**AP1**: Activator Protein 1
**TCF**: T-cell Factor
**ELK**: ETS-like protein
**STAT**: Signal Transducer and Activator of Transcription
**c-JUN**: cellular-JUN
**c-MYC**: cellular-MYC
**MyD88**: Myeloid differentiation primary response 88
**IRAK-1**: Interleukin-1 Receptor associated kinase 1
**TRAF6-INF**: Receptor Associated Factor 6
**TAB**: TAK1-binding protein
**ASK1**: Apoptosis Signal-regulating kinase 1
**TAK1**: Transforming growth factor-β-Activated kinase-1
**PI3K**: Phosphoinositide-3-kinase
**MKK-4/3/6**: MAPK kinase 4, 3 and 6
**p38α**: p38 α MAPK
**p110**: Catalytic subunit of PI3K
**T306**: Threonine 306 (phosphorylation site)
**S473**: Serine 473
TSC1/2: Tuberous sclerosis complex 1 and 2
**mTORC2**: Mechanistic Target of Rapamycin Complex 2
**Rheb**: Ras homolog enriched in brain
**p70S6K**: p70 Ribosomal S6 kinase
**4EBP-1**: Eukaryotic Translation Initiation Factor 4E-binding protein
**EIF4E**: Eukaryotic Translation Initiation Factor 4E
**MDM2**: Mouse-Double Minus 2 homolog (E3 ubiquitin protein ligase)
**XIAP**: X-linked Inhibitor of Apoptosis Protein
**FOXO**: Forkhead box O
**Bcl2**: B-cell lymphoma 2
**SARA**: SMAD Anchor for Receptor Activation
**SMAD2**: SMAD family member 2
**SMAD3**: SMAD family member 3
**SMAD4**: SMAD family member 4
**TGF-βT**: €Transforming Growth Factor-beta Receptor
**ROS**: Reactive Oxygen Species
**RNS**: Reactive Nitrogen Species
**ABC Transporter**: ATP-binding cassette transporter
**P-gp ATPase**: P-glycoprotein ATPase
**Tyr**: Tyrosine
**TFR1**: Transferrin Receptor-1
**TF**: Transferrin
**DMT1**: Divalent Metal Ion Transporter
**TCA cycle**: Tricarboxylic Acid cycle
**STEAP3**: Six Transmembrane Epithelial Antigen of the Prostate 3
**DFO**: Deferoxamine
**NCOA4**: Nuclear Receptor Coactivator 4
**GSH**: Glutathione
**DLAT**: Dihydrolipoamide Acetyltransferase
**RBX1**: RING-box Protein 1
**Cul3**: Cullin 3
**KEAP1**: Kelch-like ECH-associated Protein 1
**Nrf2**: Nuclear-Factor Erythroid 2-related Factor 2
**Smaf**: Small Maf Protein
**ARE**: Antioxidant Response Element
**GSK3β**: Glycogen Synthase Kinase 3 β
**B-TrCP**: Beta-Transducin Repeat Containing Protein
**Cul1**: Cullin 1
**Ub**: Ubiquitin
**HO-1**: Heme Oxygenase-1
**NQO-1**: NAD(P)H-Quinone Oxidoreductase 1
**GSLS**: Glycosphingolipids
**PSTs**: Protein sulfotransferase
**TAK1**: TGF-β Activated kinase 1
**IRS**: Insulin Receptor Substrate
**MK2**: Mitogen Activated Protein Kinase-Activated Protein Kinase 2
**MK3**: Mitogen Activated Protein Kinase-Activated Protein Kinase 3
**HSP27**: Heat shock protein 27
**PRAK1**: p38-Regulated/Activated Kinase 1
**MSK-1**: Mitogen-and Stress-Activated Protein Kinase 1
**ChG-8**: protein code
**CHOP/C/EBP**: Homologous Protein
**MEF**: Myocyte Enhanse Factor
**MKK2/3**: Mixed-lineage Kinase 2 and 3
**RAC**: Ras-related (3 botulinum toxin substrate)
**CDC42**: cell division cycle 42
**CD-45**: Region 45
**JAK**: Janus kinase
**PTPS**: Protein-Tyrosine Phosphatase
**SOCS**: Suppressors of Cytokine Signals
**PIAS**: Protein Inhibitors of Activated STAT
**MCL7**: Myeloid Cell Leukemia-1
**VEGF-2**: Vascular Endothelial Growth Factor Receptor-2
**PAMP**: Pathogen Associated Molecular Pattern
**DAMP**: Damage Associated Molecular Pattern
**ILIRI**: Interleukin 1 Receptor Type 1
**P2X7R**: P2X7 Receptor
**LPS**: Lipopolysaccharides
**SIRT2**: Sirtuin 2
**NLRP3**: NOD-, LRR- and Pyrin Domain-containin Protein 3
**ASC**: Apoptosis-Associated Speck-like Protein containing a CARD
**NEK-7**: NIMA-related kinase 7
**GISDMD**: Gasdermin D
**GISDMD-NT**: Gasdermin DN-terminal domain
**AKT**: Protein Kinase B
**PIP3/2**: Phosphatidylinositol (3,4,5)
**p85**: Regulatory subunit of p85 of PI3K
